# A relation of serum homocysteine and uric acid in Bosnian diabetic patients with acute myocardial infarction

**DOI:** 10.5937/jomb0-28391

**Published:** 2021-06-05

**Authors:** Marijana Marković-Boras, Adlija Čaušević, Marina Ćurlin

**Affiliations:** 1 University Clinical Hospital Mostar, Department of Laboratory Medicine, Mostar, Bosnia and Herzegovina; 2 University of Sarajevo, Faculty of Pharmacy, Department of Biochemistry and Clinical Analysis, Sarajevo, Bosnia and Herzegovina; 3 University of Mostar, Faculty of Health Studies, Mostar, Bosnia and Herzegovina

**Keywords:** diabetes mellitus type 2, homocysteine, myocardial infarction, uric acid, diajbetes melitus tip 2, homocistein, infarkt miokarda, mokraćna kiselina

## Abstract

**Background:** Coronary artery disease as a consequence of atherosclerosis is the most common cause of morbidity and mortality in type 2 Diabetes Mellitus (DM) patients. Homocysteine (HCY), as one of the risk factors, and uric acid (UA) as the most common antioxidant in serum have their roles in the processes of inflammation and atherogenesis, which underlie the pathogenesis of acute myocardial infarction (AMI). The effect of HCY in cardiovascular disease is thought to be manifested primarily through oxidative damage, implying a potential correlation between the HCY level and antioxidant status. Since the data related to the diagnostic significance of both HCY and UA in diabetic patients with AMI are conflicting, and so far not reported in Bosnian patients, this research aimed to examine the association of HCY and UA levels with glomerular filtration rate (eGFR) and explore the pathophysiological significance of these data in Bosnian diabetic patients with AMI.

**Methods:** This prospective research included 52 DM type 2 patients diagnosed with AMI. Blood samples were taken on admission and used for biochemical analysis. Results of the biochemical analyses were statistically analysed.

**Results:** Elevated HCY and UA levels were observed in diabetic patients. Females have higher HCY compared to males. A positive correlation was revealed between HCY and UA and was confirmed with different HCY levels in subgroups with different UA level. A negative correlation was observed between UA and HbA1c, as well as between both HCY and UA with eGFR.

**Conclusions:** These results contribute to the clarification of the biochemical mechanisms characteristic in AMI patients with DM. According to these results, we believe that joint measurement of HCY and UA could enable a better assessment of the prognosis for this group of patients. This kind of assessment, as well as regression analysis, can identify high-risk patients at an earlier stage when appropriate interventions can influence a better outcome in such patients.

## Abstract (sr)

**Uvod:** Bolest koronarnih arterija kao posledica ateroskleroze je najčešći uzrok morbiditeta i mortaliteta kod pacijenata sa dijabetesom melitus tipa 2 (DM). Homocistein (HCY), kao jedan od faktora rizika, i mokraćna kiselina (UA), kao najčešći antioksidans u serumu, imaju svoju ulogu u procesima upale i aterogeneze koji su u osnovi patogeneze akutnog infarkta miokarda (AMI). Smatra se da se efekat HCY na kardiovaskularne bolesti manifestuje prvenstveno kroz oksidativno oštećenje, implicirajući potencijalnu korelaciju između koncentracije HCY i antioksidativnog statusa. Budući da su podaci koji se odnose na dijagnostički značaj HCY i UA kod dijabetičara sa AMI oprečni, i da do sada nisu nisu bili dostupni za pacijenate iz Bosne i Hercegovine, cilj ovog istraživanja je bio da se ispita povezanost koncentracije HCY i UA sa stopom glomerularne filtracije (eGFR) i istraži patofiziološki značaj ovih podataka kod pacijenata iz Bosne i Hercegovine koji su dijabetičari sa AMI.

**Metode:** Ovim prospektivnim istraživanjem obuhvaćena su 52 pacijenta sa dijabetesom tipa 2 kojima je dijagnostikovan AMI. Uzorci krvi su uzeti prilikom prijema pacijenata i korišćeni su za biohemijsku analizu. Rezultati biohemijskih analiza su statistički analizirani.

**Rezultati: **Primećeni su povišene koncentracije HCY i UA kod dijabetičara. Žene imaju veći HCY u poređenju sa muškarcima. Otkrivena je pozitivna korelacija između HCY i UA i potvrđena je različitim koncentracijama HCY u podgrupama sa različitim nivoom UA. Negativna korelacija je primećena između UA i HbA1c, kao i između HCY i UA sa eGFR.

**Zaključak: **Ovi rezultati doprinose pojašnjenju biohemijskih mehanizama karakterističnih za AMI pacijente sa DM. Prema ovim rezultatima, verujemo da bi zajedničko merenje HCY i UA moglo da omogući bolju procenu prognoze za ovu grupu pacijenata. Ova vrsta procene, kao i regresiona analiza, mogu identifikovati visoko rizične pacijente u ranijoj fazi kada odgovarajuće intervencije mogu uticati na bolji ishod kod takvih pacijenata.

## Introduction

Acute myocardial infarction (AMI), occurs as a result of atherosclerotic plaque rupture and formation of thrombus. Myocardial tissue becomes inflamed and necrotic; it loses contraction and impulse conducting ability with the net result of decreased oxygen distribution and irreversible damage to the heart muscle [1].

Cardiovascular disease (CVD) and AMI are the most common causes of morbidity and mortality in patients with Diabetes Mellitus (DM) [2]. They have two to four times higher mortality due to AMI and stroke compared to patients of the same age without DM. The mortality rate is between one and three times higher in males and between two and five times higher in females than the control group without diabetes [3]. In the post-infarction period, mortality is significantly more frequent in patients with DM.

Novel studies of CVD risk factors in patients with diabetes indicate the diagnostic value of elevated homocysteine (HCY) and uric acid (UA) levels. An increased level of HCY in AMI patients compared to the group of healthy subjects was observed in the previous research [4] [5], while in some [6], such association was not observed. Results of previous research related to the possible relationship between HCY and DM show a high degree of inconsistency. Some authors reported an association between increased HCY level in DM type 2 and insulin resistance [7] as well as an increased risk of vascular disease, while others found a decreased level of HCY in diabetics [8] or no difference in HCY level between diabetics and healthy subjects [9]. It is believed that hyperhomocysteinemia may be the cause and/or the consequence of insulin resistance.

So far, it has been shown that serum UA levels are elevated in patients with AMI, involving categories belonging to patients with systemic hypertension and those with DM, when compared to healthy individuals. Diabetic patients who are hyperuricemic appear to be at increased risk of developing diabetic complications, especially renal and cardiovascular [10]. Previous studies have reported that a high concentra-tion of UA is also a strong marker of an unfavourable prognosis of moderate to severe heart failure and CVD. Namely, hyperuricemia has a critical impact on poor outcome in AMI patients. Since the effect of both HCY and UA in CVD is thought to be primarily related to oxidative damage on the vascular endothelium, causing alterations in the vasodilatory properties of endothelial cells due to disturbances in nitric oxide (NO) bioavailability and scavenging [11], it was not a surprise to observe that joint effect of hyperhomocysteinemia and hyperuricaemia has a stronger effect on changes on the vascular endothelium [12].

Although HCY and UA have many similarities, their relation is not quite clear in diabetic patients with AMI. A detailed literature review indicated that at least so far there have been no studies trying to link the HCY level and compounds with antioxidant activity level, which are measured in routine clinical practice in percutaneous coronary intervention (PCI). Therefore, the aim of this research is to elucidate the relation between laboratory markers, mainly HCY and UA levels, to correlate these results to glomerular filtration rate (eGFR) and to determine their significance in diabetic patients with AMI.

## Materials and Methods

This prospective research included 52 DM type 2 patients diagnosed with AMI. Patients were admitted to the Department of Cardiology at the University Clinical Hospital of Mostar, Bosnia and Herzegovina, in the period between October 2016 and January 2019. Patients were previously diagnosed with DM type 2 based on ADA recommendations [13].

Study design, methods and statistical analysis are similar to our previous research published in August 2018 [14]. The AMI diagnosis was made by a cardiologist on the basis of at least two of the three WHO criteria [1]. All patients were diagnosed with STEMI myocardial infarction, before performance of PCI as a treatment method. STEMI was confirmed by electrocardiogram (ECG). Detailed medical history, including usual CVD risk factors, such as hypertension and smoking, was taken for each patient. Hypertension was defined as systolic arterial pressure ≥140 mmHg and/or diastolic arterial pressure ≥90 mmHg or as the use of antihypertensive drugs. Smoking was defined as daily consumption of at least one cigarette. Exclusion criteria were pregnancy, the use of antiepileptic, contraceptive therapy, cancer, and vitamin B12 supplementation in the last 6 months.

All participants in this research were informed about the details of the study. The research did not affect the treatment and hospitalisation duration of patients. It was done in accordance with ethical recommendations and practices of Mostar University Clinical Hospital and the Declaration of Helsinki.

Due to the simpler interpretation of possible statistically significant results, according to previous studies [14] [15] patients involved were divided by UA in tertiles and according to gender. In the correlation analysis, patients were stratified according to age, gender, smoking, hypertension, UA, HCY, HbA1c and eGFR level as well.

On admission, after ECG, and before the PCI, venous blood samples were taken into the vacuum tubes. A serum sample used to determine the UA and HCY level was taken with one anticoagulant-free 7.5 mL test tube (Sarstedt, Germany). Whole blood sample for HbA1c assay was taken in a single 2.6 mL test tube with EDTA as an anticoagulant (Sarstedt, Germany). Residual parts of the samples were taken for research after the completion of routine treatment ordered by a cardiologist. The level of the HCY (mmol/L) was measured in the serum by non-competitive immunoassay method on Architect ci8200 Integrated System analyser (Abbot, Ilinois, USA). The UA level was measured according to the standardised IFCC (International Federation of Clinical Chemistry) photometric method (uricase-POX) on the AU680 analyser (Beckman Coulter, California, USA). HbA1c was measured on the same analyser according to standardised IFCC method-immunoturbidimetry. The statistical analysis was performed by SPSS (SPSS Inc., Chicago, IL, U.S.A.). The Kolmogorov-Smirnov test tested an assessment of the normality of data. Correlations were tested by the nonparametric Spearman correlation. The nonparametric Mann-Whitney U test tested the differences between UA and HCY level in different subgroups of patients. The comparison of HCY and UA with respective reference ranges was performed by one-sample t-test. Kruskal-Wallis and χ^2^ tests were used in cases of comparison of more than two groups (stratification of patients according to UA level in tertiles). P<0.05 was considered statistically significant.

## Results

As represented in Table 1, this study included 52 diabetic patients diagnosed with AMI. The median age was 64.20±9.57 years; 60.75±6.98 and 71.81±8.81 years for males and females, respectively (p<0.001). It consisted of 40 male (77%) and 12 female (23%) patients.

Hypertension was equally distributed between males and females (65% [16] and 75% [9], respectively). The prevalence of smoking was 46% [17]; with 55% [18] and 17% [2] in males and females, respectively. Further analyses revealed lower HCY level in males (12.99±4.22 µmol/L) compared to females (16.94±5.30 µmol/L) (p=0.042). Females had a lower eGFR (p=0.004) level as well. A statistically significant difference in other measured parameters was not observed (Table 1).

**Table 1:** Demographic characteristics and biochemical parameters level ^1^ t-test, ^2^ χ^2^ test,^ 3^Mann-Whitney U

Variable (No; %)	All patients (n=52)	Males (n=40)	Females (n=12)	p
Age (years) (mean ±SD)	64.20±9.57	60.75±6.98	71.81±8.81	<0.001^1 ^
Gender (males)	-	40 (77)	12 (23)	<0.001^2^
Smoking (yes)	24 (46)	22 (55)	2(17)	
Hypertension (yes)	36(69)	26 (65)	9 (75)	
Parameter (Mean ±SD)				
Homocysteine, mmol/L	14.29±5,21	12.99±4.22	16.94±5.30	0.042^3^
Uric acid, mmol/L	335.46±112,12	341.89±116.93	311.63±80.87	
HbA1c, %	7.39±1.61	7.51±1.64	7.06±1.57	
eGFR-60mL/min/1.73 m^2^	72.54±15,15	74.13±13.94	62.37±13.56	0.004^1^

**Figure 1:**
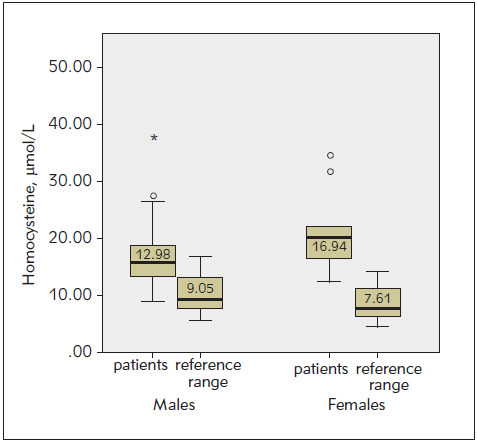
Comparison of homocysteine level with reference ranges for male and female patients. The graphs show the scatter of the results within the group. The thick black line in the middle is the median, inside the coloured area are the results between 25% (lower edge) and 75% (upper edge of the rectangle), i.e. 50% of all results, while the horizontal end lines indicate the range of results covering the largest and smallest results. They do not go beyond the framework of distribution (they are not outliers or extreme results). (p<0.001 one-sample t-test)

**Figure 2:**
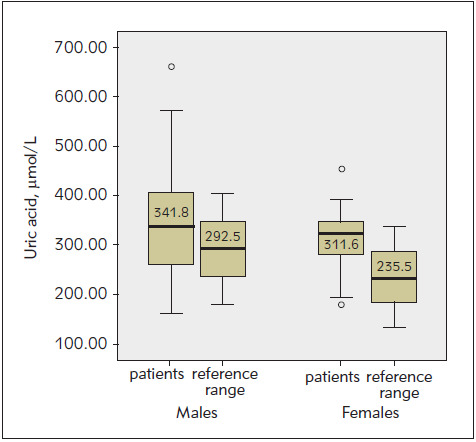
Comparison of UA level in gender-dependent subgroups with reference ranges. The graphs show the scatter of the results within the group for each variable. A thick black line in the middle of the rectangle indicates the mean value (p<0.001 t-test (one sample))

In order to compare HCY and UA levels with the healthy population, results were compared with gender-dependent reference ranges (Figure 1 and Figure 2). A significantly increased HCY level was observed in both male (p<0.001) and female (p<0.001) patients compared to »normal« values within the reference range. The UA level was also compared with the gender-dependent reference range values. A significant increase in UA level was observed in both males (p<0.001) and females (p<0.001) compared to the »normal« values within the reference range.

The analysis of subgroups obtained by distribution according to the level of UA in tertiles revealed a reduced level of HCY in the first tertile, compared to the second and third (p=0.030) (Table 2). eGFR shows a strong association with UA. Patients with lower eGFR values had significantly elevated serum UA level (p=0.003). The incidence of hypertension was significantly higher in the third tertile with the highest level of UA (88% [15]) compared to the first (38% [7]) and second (76% [13]) (p=0.008) (Table 2).

**Table 2:** Distribution of patients according to Uric acid ^1^Kruskal-Wallis, ^2^ANOVA, ^3^ χ^2^ test

Uric acid (mmol/L)				
Variable (No; %)	Group 1 <272.0 N=18	Group 2 272.0-356.0 N=17	Group 3 >356.0 N=17	P
Age (years) (mean ±SD)	63.27±8,66	65.93±9.51	61.18±6.61	0.283^2^
Gender (males)	15 (83%)	10 (59%)	14 (82%)	0.185^3^
Smoking (yes)	6 (33%)	10 (58%)	7 (41%)	0.519^3^
Hypertension (yes)	7 (38%)	13 (76%)	15 (88%)	0.008^3^
Parameter (Mean ±SD)				
Homocysteine, mmol/L	12.09±4.42	17.17±6.62	15.65±5.79	0.030^1^
Uric acid, mmol/L	229.37±33.93	314.50±24.30	464.43±86.17	<0.001^1^
HbA1c, %	8.14±1.89	7.00±1.04	7.05±1.61	0.091^1^
eGFR-60mL/min/1,73 m^2^	81.55±14.17	68.77±10.99	62.57±18.50	0.003^2^

Correlation analyses in different subgroups are represented in Table 3. In simple correlation analyses between UA and HCY, a positive correlation occurred (rho=0.298). The same correlation was noticed in patients with eGFR value lower than the median value (rho=0.464). HCY shows negative correlation with eGFR (rho=-0.338). A correlation occurred in the third tertile of UA (rho=-0.574) and the subgroup with eGFR value lower than the median value (rho=-0.463) as well. A negative correlation was observed between eGFR and UA (rho=-0.525). A correlation was observed in the third tertile of UA (rho=-0.509) and an elevated HCY group (rho=-0.672). The correlation was strong in the elderly (rho=-0.799), as well as in patients with decreased eGFR (rho=-0.513) and was present in nonsmokers and patients with hypertension as well (rho=-0.644 and rho=-0.404, respectively). The correlation was moderate in both males (rho=-0.556) and females (rho=-0.685). A weak negative correlation was observed between UA and HbA1c (rho=-0.322). In low HbA1c subgroup, a weak positive correlation occurred as well (rho=0.453). All mentioned correlations were at the level of significance p<0.05 (Table 3).

**Table 3:** The correlation analysis *median value 13.56 mmol/L, ** median value 6.80 %, ***median value 70.6 60 mL/min/1.73 m^2^

Patient group (No)	Spearman correlation (p)			
	HCY/UA	HCY/eGFR	UA/eGFR	UA/HbA1c
All patients (52)	rho=0.298 p=0.038	rho=-0.338 p=0.023	rho=-0.525 p<0.001	rho=-0.322 p=0.024
Age (years)				
<55 (10)	p=0.139	p=0.966	rho=-0.565/ p=0.356	p=0.460
55-65 (23)	p=0.480	p=0.145	rho=-0.799/ p=0.009	p=0.519
>65 (19)	p=0.062	p=0.383	p<0.001	p=0.180
Gender				
Males (40)	p=0.101	p=0.212	rho=-0.556/ p=0.001	p=0.090
Females (12)	p=0.110	p=0.310	rho=-0.685/ p=0.029	p=0.055
Smoking				
Yes (24)	p=0.345	p=0.192	p=0.078	p=0.790
No (28)	p=0.715	p=0.220	rho=-0.644/ p=0.002	p=0.376
Hypertension				
Yes (30)	p=0.216	p=0.140	rho=-0.404/ p=0.030	p=0.353
No (22)	p=0.260	p=0.943	p=0.655	p=0.186
Homocysteine				
<median value* (26)	p=0.300	p=0.816	p=0.134	p=0.083
>median value* (26)	p=0.737	p=0.113	rho=-0.672/ p=0.001	p=0.265
Uric acid				
<272.0 mmol/L (18)	p=0.713	p=0.899	p=0.703	p=0.271
272.0-356.0 µmol/L (17)	p=0.077	rho=-0.574/p=0.550	rho=-0.509/ p=1.00	p=0.795
>356.0 mmol/L (17)	p=0.602	p=0.020	p=0.044	p=0.473
HbA1c				
<median value** (26)	p=0.842	p=0.099	p=0.173	rho=0453/ p=0.026
>median value** (26)	p=0.134	p=0.416	p=0.094	p=0.674
GFR				
<median value***(26)	rho=0.464/ p=0.022	rho=-0.463/p=0.023	rho=0.513/ p=0.010	p=0.453
>median value***(26)	p=0.819	p=0.186	p=0.104	p=0.195

## Discussion

Descriptive characteristics represented in Table 1 indicate a higher incidence of males compared to females in our study, i.e. the results of the research were obtained predominantly on the male population. The incidence of 77% of males is consistent with the previous research in which female patients were identified as less frequent but also as patients with a poorer prognosis after AMI [19].

Smoking, which is previously recognised as a significant and independent risk factor for all-cause, cardiovascular, and noncardiovascular mortality and fatal and non-fatal coronary heart disease and congestive heart failure is represented in 46% patients of this research. Smoking is a risk factor for mortality and coronary heart disease in patients with hypertension as well as with DM. However, there is no evidence that the relative risk of smoking is higher in people with diabetes than in people without DM. Because DM alone is a risk factor for morbidity and mortality, the absolute risk of smoking is usually higher in people with diabetes than in people without DM [20]. The positive association between blood pressure, which is in this research represented in 69% of patients, and CVD was well documented in recent studies [21] [22]. Smoking is also associated with blood pressure level and CVD mortality [23]. In addition to the above, recent studies revealed a significant relationship between the combined effects of smoking and blood pressure on CVD mortality [20].

In this research, HCY level is higher in females. This implies a contribution of DM to the elevation of HCY level in this population, which confirms the fact that females with diabetes are the group with the highest risk of developing CVD, and hospital mortality in female patients with AMI compared to males is significantly increased [24]. In our study, female patients were older than males, which is in line with research Cui et al. [25]. The females were postmenopausal when the protective vasodilating effect of endoge-nous estrogen is weak [18], which partly explains the increased risk observed in these patients. The action of estrogen in CVD in females is manifested through an increase in NO release leading to vasodilatation, in the regulation of prostaglandin synthesis and inhibition of epithelial smooth muscle cell proliferation. Due to the failure of these mechanisms in postmenopause, endothelial dysfunction occurs, with lipid deposition on the endothelium of blood vessels, ultimately favouring the development of atherosclerosis [26].

The available data in the literature on the association between CVD and hyperhomocysteinemia are contradictory; some suggest an association [11]. while others do not, and explain it by the high HCY level in the general population [6]. Contradictory results may be due to different selection of research participants, i.e. different inclusion and exclusion criteria in the study, different number of participants, methods used, genetic basis and different eating habits [17]. Genetic basis, sufficient intake of vitamins B6, B12 and folic acid, as well as preserved renal function, are factors that directly affect HCY level [11] and should all be considered during assessment in any case.

Hyperuricemia is also known to be a critical factor in the development of CVD in the elderly, which is why early detection of UA can identify and prevent it. UA exerts its effects in different ways, which is why it can be associated with the occurrence and development of the disease [27]. UA levels are elevated in patients with AMI compared with normal healthy individuals. UA levels are elevated in systemic hypertension, and diabetes mellitus patients with AMI [16], e.g. patients with hyperuricemia have an increased risk of developing type 2 DM [28]. The described increased UA level in patients with AMI compared to the healthy control group [29] is confirmed in this research. Serum UA level in the males and females are higher than the reference range values to which a positive antioxidant effect is usually associated. In epidemiological and clinical studies, the effect of UA has been observed not only as an indicator of cardiovascular risk but also as a cause of endothelial dysfunction. Endothelial dysfunction is defined as a condition of impaired vascular homeostasis due to a disturbed balance of endothelial vasodilators and vasoconstrictors, leading to the progression of atherosclerosis.

In this research, HCY level, as a marker of oxidative stress was correlated with UA level, which has antioxidant activity in the body. The association between HCY and UA can be easily explained by hydrolysis of S-adenosyl HCY when adenosine is formed, which is further degraded to UA [17]. This implies that every increase in HCY will result in an increase in UA. This was observed in our research on diabetic patients, but also healthy subjects, cardiovascular patients as well as stroke patients [30]. In our study, a positive correlation between HCY and UA was observed. A review of biochemical characteristics in patients divided according to the UA level in tertiles revealed a significant difference in HCY level, i.e. patients with low UA level showed significantly reduced HCY level, which further confirms this connection. These results are consistent with the previous study [12]. A study by Kiseljaković et al. [31] revealed the same association in patients with atherosclerotic vascular disease and healthy subjects. In addition to the proposed mechanisms, HCY increases arterial stiffness [32], reduces the possibility of methylation causing endothelial dysfunction as well as vascular smooth muscle cell proliferation, oxidative stress, NF-kB activation, inflammation, and inhibition of NO synthesis in the endothelium [17]. It was also confirmed that the combined effect of hyperhomocysteinemia and hyperuricaemia has a stronger effect on these epithelial changes [12], suggesting the need to observe the synergistic action of HCY and UA in CVD in patients with type 2 DM.

The analysis of parameters distributed in tertiles according to UA level revealed a strong association of elevated UA level with a higher frequency of hypertension. Several previous epidemiological studies confirmed this association [33]. However, it is still unclear, whether UA is an independent causal factor, a mediator, or just an indicator of the development of hypertension. Proposed mechanisms that are mainly based on research data obtained from animal models and cell cultures include endothelial damage, vascular inflammation, and activation of the reninangiotensin system. Briefly, hypertension results from the action of UA, which causes vasoconstriction in the kidney due to decreased NO synthesis in the endothelium, with activation of the renin-angiotensin system. Independently of these changes, UA causes microvascular damage to the kidneys over time (histologically similar to atherosclerosis), which further encourages the development of hypertension [33]. Discovering the role of UA in the development of hypertension is complex because both UA and blood pressure are associated with renal function as well as other common metabolic disorders [33] [34]. Therefore, we analysed whether UA and HCY are associated with glomerular filtration rate (eGFR). The association of UA with the renal function was confirmed by a strong negative correlation between UA and eGFR. Distribution of groups according to UA level in tertiles different eGFR level were also observed in the obtained subgroups. In this study, decreased eGFR values were observed in female subjects. Female gender, low eGFR, and high creatinine level on admission in coronary units are important prognostic factors for impaired cardiac function and increased mortality in the post-infarction period and can be used to assess risk in patients with AMI [35]. In several smaller clinical studies, the effect of lowering UA on lowering blood pressure in patients with hypertension was observed [33] [36]. This, however, suggests UA as a causal factor in the development of hypertension, especially in the early stages before the onset of significant endothelial damage [37]. A negative correlation of HCY with eGFR was observed, which further confirms the association with the preservation of renal function. A decrease in eGFR is thought to cause a decrease in HCY secretion. Hyperhomo cystei nemia was suggested as an important pathogenic factor leading to glomerular injury, dysfunction, and sclerosis [32]. Increased oxidative stress and decreased antioxidant defence function caused by hyperhomocysteinemia were proven to be associated with the renal function [38]. The observed negative correlation with eGFR suggests the importance of preserving renal function in the regulation of HCY and UA level.

Negative correlations were also observed between UA and HbA1c. A negative correlation is considered to indicate the existence of hyperinsulinemia in these patients, and UA is considered a good indicator of blood glucose level. However, an increase in UA compared with an increase in glucose is a characteristic of a healthy population and patients with prediabetes, but in type 2 DM, a decrease in UA was observed with an increase in HbA1c, which was found in this study as well. The reason for the reverse association is still unclear, but insulin is thought to be a factor influencing this association [25]. Serum UA level will increase with an increased insulin level in patients with diabetes [39]. Insulin causes the activation of the pentose phosphate pathway, which stimulates the biosynthesis and transformation of purines, as well as the synthesis of UA. At the same time, insulin causes an increase in UA reabsorption in the kidneys by stimulating the urate anion transporter on the proximal tubules, which ultimately causes an increase in serum level [40]. At the same time, insulin causes a decrease in serum glucose level, and thus affects the levels of both parameters.

According to the results of our study, both HCY and UA are factors of atherosclerosis. Hence their action in the body should not be observed independently because their metabolism, as well as the effect on the cardiovascular system, show significant overlaps. Also, both of these markers can be used as markers of renal dysfunction in these patients.

Our study has several limitations. It is a singlecentre study with a relatively small group of patients. Control subjects without diabetes were not included in our study. Plasma levels of vitamin B and folate, both of which influence the plasma level of HCY, were not measured.

In conclusion, this study indicates that a combined assessment of HCY and UA, as well as a regression analysis, can identify high-risk patients at an earlier stage, and appropriate interventions can influence a better outcome in such patients. Identification of hyperhomocysteinemia as a risk factor, especially in female patients, is of great importance for the diabetic population primarily because of primary and secondary prevention in these patients involving the use of a number of vitamins such as folic acid, vitamin B12 and pyridoxine that directly affect serum HCY level. Therefore, further research studying the impact of the use of these vitamins on the reduction of serum HCY level in the diabetic population of Bosnia and Herzegovina would be of multiple importance. Also, the use of HCY as a marker in prediabetes and diabetes would contribute to the establishment of sub-clinical CVD and suggest the need to take preventive measures before a possible occurrence of infarction or stroke.

## Conflict of interest statement

The authors have no conflicts of interest to declare. The authors give consent to the submission and publication of the work. Authors disclose no relationship to any organization or industrial manufacture in any material discussed.

## List of abbreviations

DM, diabetes mellitus; CVD, cardiovasculardisease; AMI, acute myocardial infarction; STEMI, ST-segmentelevation myocardial infarction; HCY, homocysteine; UA,uric acid; PCI, percutaneous coronary intervention; NO, nitricoxide; ECG, electrocardiogram; IFCC, International Federationof Clinical Chemistry.
